# Psilocybin with psychological support for treatment-resistant depression: six-month follow-up

**DOI:** 10.1007/s00213-017-4771-x

**Published:** 2017-11-08

**Authors:** R. L. Carhart-Harris, M. Bolstridge, C. M. J. Day, J. Rucker, R. Watts, D. E. Erritzoe, M. Kaelen, B. Giribaldi, M. Bloomfield, S. Pilling, J. A. Rickard, B. Forbes, A. Feilding, D. Taylor, H. V. Curran, D. J. Nutt

**Affiliations:** 1 Psychedelic Research Group, Centre for Neuropsychopharmacology, Division of Brain Sciences, Faculty of Medicine, Imperial College London, London, UK; 2 South London and Maudsley NHS Foundation Trust, London, UK; 3 The Institute of Psychiatry, Psychology and Neuroscience, King’s College London, London, UK; 4 South West London and St George’s Mental Health NHS Trust, London, UK; 5 Division of Psychiatry, University College London and Clinical Psychopharmacology Unit, University College London, London, UK; 6 Clinical Psychology and Clinical Effectiveness, University College London, London, UK; 7 Barts Health Pharmaceuticals, Barts Health NHS Trust, the Royal London Hospital, London, UK; 8 Institute of Pharmaceutical Science, King’s College London, London, UK; 9 The Beckley Foundation, Beckley Park, Oxford, UK; 10 Pharmacy and Pathology, South London and Maudsley NHS Foundation Trust, London, UK; 11 Clinical Psychopharmacology Unit, University College London, London, UK

**Keywords:** Serotonin, 5-HT2AR, Depression, Treatment-resistant depression, Psilocybin, Psychedelic, Mood, Hallucinogen, Psychotherapy

## Abstract

**Rationale:**

Recent clinical trials are reporting marked improvements in mental health outcomes with psychedelic drug-assisted psychotherapy.

**Objectives:**

Here, we report on safety and efficacy outcomes for up to 6 months in an open-label trial of psilocybin for treatment-resistant depression.

**Methods:**

Twenty patients (six females) with (mostly) severe, unipolar, treatment-resistant major depression received two oral doses of psilocybin (10 and 25 mg, 7 days apart) in a supportive setting. Depressive symptoms were assessed from 1 week to 6 months post-treatment, with the self-rated QIDS-SR16 as the primary outcome measure.

**Results:**

Treatment was generally well tolerated. Relative to baseline, marked reductions in depressive symptoms were observed for the first 5 weeks post-treatment (Cohen’s *d* = 2.2 at week 1 and 2.3 at week 5, both *p* < 0.001); nine and four patients met the criteria for response and remission at week 5. Results remained positive at 3 and 6 months (Cohen’s *d* = 1.5 and 1.4, respectively, both *p* < 0.001). No patients sought conventional antidepressant treatment within 5 weeks of psilocybin. Reductions in depressive symptoms at 5 weeks were predicted by the quality of the acute psychedelic experience.

**Conclusions:**

Although limited conclusions can be drawn about treatment efficacy from open-label trials, tolerability was good, effect sizes large and symptom improvements appeared rapidly after just two psilocybin treatment sessions and remained significant 6 months post-treatment in a treatment-resistant cohort. Psilocybin represents a promising paradigm for unresponsive depression that warrants further research in double-blind randomised control trials.

**Electronic supplementary material:**

The online version of this article (10.1007/s00213-017-4771-x) contains supplementary material, which is available to authorized users.

## Introduction

Psilocybin is a naturally occurring plant alkaloid that is being increasingly researched as treatment for a range of different psychiatric disorders (Carhart-Harris and Goodwin 2017). Four separate trials have reported improvements in depressive symptoms after psilocybin-assisted psychotherapy (Griffiths et al. 2016; Ross et al. 2016; Grob et al. 2011; Carhart-Harris et al. 2016), including one in which ‘treatment-resistant depression’ was the primary criterion for inclusion (Carhart-Harris et al. 2016). Psilocybin has shown promise in the treatment of obsessive compulsive disorder (Moreno et al. 2006), alcohol (Bogenschutz et al. 2015) and tobacco addiction (Johnson et al. 2014) and anxiety related to terminal diagnoses (Griffiths et al. 2016; Ross et al. 2016; Grob et al. 2011). Treatment procedures typically involve psychological preparation prior to one or two therapist-supported drug sessions followed by psychological integration. Using a consistent model (i.e. involving appropriate psychological support), sustained improvements in well-being in healthy individuals were observed after a single dose of psilocybin in a double-blind design incorporating an active placebo (Griffiths et al. 2008).

Studies involving other serotonergic psychedelics combined with psychological support have found similarly promising outcomes: Sustained reductions in end-of-life anxiety were observed after LSD-assisted psychotherapy (Gasser et al. 2014), and reduced depressive symptoms were seen after ayahuasca in patients with ‘recurrent depression’ (Osorio Fde et al. 2015; Sanches et al. 2016). Naturalistic, observational studies of ayahuasca support its long-term well-being promoting and anti-addiction properties (Thomas et al. 2013; Bouso et al. 2012) and a recent population survey found lower rates of suicidality and psychological distress in association with psychedelic drug use (Hendricks et al. 2015)—an anomalous association for a drug of potential misuse. Drug experts and users have consistently rated psilocybin as the least harmful and potentially ‘most beneficial’ drug of potential misuse (Carhart-Harris and Nutt 2013; van Amsterdam et al. 2015)—although the influence of *context* (e.g. expectations and environmental factors) on potential harms and benefits has been much emphasised (Hartogsohn 2016; Carhart-Harris et al., in review). Further evidence favouring the therapeutic potential of psychedelics can be found in literature documenting the extensive research carried out with these compounds in the mid-twentieth century, e.g. two relevant meta-analyses have found positive safety and efficacy data for LSD for alcohol dependence (Krebs and Johansen 2012) and mood disorders (Rucker et al. 2016). See Carhart-Harris and Goodwin (2017) for a review of historical and recent trials with psychedelics.

Like all serotonergic psychedelics, psilocybin initiates its characteristic effects via serotonin 2A receptor (5-HT2AR) agonism (Vollenweider et al. 1998). 5-HT2AR signalling has been associated with better responses to conventional antidepressants (Qesseveur et al. 2016; Petit et al. 2014), and preclinical work indicates that 5-HT2AR signalling may mediate (at least some of) the therapeutic effects of SSRIs (Nic Dhonnchadha et al. 2005; Buchborn et al. 2014). Paradoxically, 5-HT2AR antagonists have been found to augment the antidepressant effects of SSRIs (Ostroff and Nelson 1999) and many effective antidepressant augmentation medications have 5-HT2AR antagonist properties (Carpenter et al. 1999). This paradox implies that 5-HT2AR agonism *and* antagonism can achieve consistent ends, in terms of alleviating depressive symptoms, but via different mechanisms (see Carhart-Harris et al. (2017) and Carhart-Harris and Nutt (2017) for a relevant discussion).

The present report documents an extension to our recently published pilot study assessing psilocybin with psychological support for treatment-resistant depression. The number of patients treated was increased from 12 to 20 and the follow-up period extended from 3 to 6 months.

## Methods

### Approvals and drug source

This clinical trial received a favourable opinion from the National Research Ethics Service (NRES) London-West London, was sponsored and approved by Imperial College London’s Joint Research and Complication Organisation (JRCO), was adopted by the National Institute of Health Research (NIHR) Clinical Research Network (CRN) and was reviewed and approved by the Medicines and Healthcare products Regulatory Agency (MHRA). A Home Office Licence for storage and dispensing of Schedule One drugs was obtained. Psilocybin was obtained from THC Pharm (Frankfurt) and formulated into the investigational medicinal product (5 mg psilocybin in size 0 capsules) by Guy’s and St Thomas’ Hospital’s Pharmacy Manufacturing Unit (London, UK).

### Study design

This was an open-label feasibility study in 20 patients with treatment-resistant depression. Treatment involved two oral doses of psilocybin (10 and 25 mg), 7 days apart. The primary outcome was mean change in the severity of self-reported (SR) depressive symptoms (measured primarily with the 16-item Quick Inventory of Depressive Symptoms, QIDS-SR16) from baseline to specific time points after the high-dose psilocybin session (henceforth referred to as ‘post-treatment’). QIDS-SR16 ratings were collected 1–3 and 5 weeks and 3 and 6 months post-treatment, with 5 weeks post-treatment regarded as the primary endpoint. BDI (depression) and STAI (anxiety) ratings were collected at 1 week and 3 and 6 months. SHAPS (anhedonia) was collected at 1 week and 3 months and HAM-D (depression, clinician-administered) and GAF (global functioning, clinician administered) ratings were collected at 1 week only. These secondary measures were collected to enable comparisons to be made with other studies that use the same measures. For this reason and since they were highly correlated with the primary outcome measure, we chose not to correct for their use. A revised *α* of 0.05/6 = 0.0083 for the six post-treatment QIDS-SR16 contrasts vs baseline was used however.

### Trial procedures

Full details of trial procedures can be found in Carhart-Harris et al. (2016). Briefly, patients contacted the study team after which a telephone screen was organised with the main study psychiatrist. After checking eligibility criteria, candidates were invited for a screening visit at the Imperial Clinical Research Facility (ICRF) at the Hammersmith Hospital. This comprised of informed consent, documenting mental and physical health backgrounds, a psychiatric interview (MINI-5) to confirm diagnosis, physical examination, routine blood tests, ECG, urine test for drugs of abuse and pregnancy where relevant, a breathalyser and the completion of baseline assessments.

The main inclusion criteria were as follows: unipolar major depression of at least moderate severity (16+ on the 21-item HAM-D) and no improvement despite two courses of pharmacologically distinct antidepressant medications for an adequate duration (6 weeks minimum) within the current episode. Main exclusion criteria were as follows: a current or previously diagnosed psychotic disorder or an immediate family member with a diagnosed psychotic disorder.

Patients’ mental health histories were confirmed with their GP or psychiatrist prior to study entry. With the exception of patient 2 (Table 1), eligible patients medicated with an antidepressant were advised to stop this for the trial, to avoid suspected attenuation of psilocybin’s effects (Bonson et al. 1996). This was done in a tapered manner under careful supervision from the study psychiatrist. Washout occurred over at least 2 weeks prior to study entry, with the exception of patient 6, who stopped tramadol use only after the first psilocybin session (when the tramadol use was discovered).

**Table 1:** Baseline characteristics and demographics: SSRI = selective serotonin reuptake inhibitor, SNRI = serotonin-noradrenaline reuptake inhibitor, NDRI = noradrenaline-dopamine reuptake inhibitor, NSSRI = noradrenaline and specific serotonin reuptake inhibitor, MAOI = monoamine oxidase inhibitor, Na + channel blocker = sodium channel blocker (e.g. lithium), TCA = tricyclic antidepressant, SARI = serotonin antagonist and reuptake inhibitor (e.g. trazodone), DRI = dopamine reuptake inhibitor. CBT = cognitive behavioural therapy, MBT = mindfulness CBT, CNT = cognitive narrative therapy, GT = group therapy, CS = counselling, JA = Jungian analysis

Number	Sex	Age (years)	Ethnicity	Employment status	Illness duration (years)	QIDS-16	BDI	HAM-D	STAI	Past meds	Past psychotherapy	Education	Weekly alcohol	Previous psilocybin
1	Female	43	Black	Employed	30	19	36	19	72	SSRI (two), SNRI (two), NDRI, NSSRI, MAOI	None	Masters	1	0
2	Male	40	Hispanic	Unemployed	25	20	33	28	76	SSRI (two), SNRI, NDRI, NSSRI, Na + channel blocker (two), ketamine, TCA	CNT	Masters	0	0
3	Male	37	White	Employed	17	22	22	18	63	SSRI (two), SNRI	CBT, GT	College post A-levels	0	0
4	Female	30	White	Studying	10	14	26	18	67	NDRI, NSSRI	CBT	Postgrad	0	1
5	Male	34	White	Unemployed	12	19	38	25	71	SSRI (three), TCA	CBT, MBT	Degree	0	0
6	Female	57	White	Unemployed	29	19	39	23	78	SSRI (four), SNRI, SARI	CS	Degree	2	2
7	Male	52	White	Unemployed	27	18	33	22	57	TCA, SARI	CS, MBT	GCSE	0	3
8	Female	37	White	Employed	17	19	39	17	71	SSRI (two), TCA	CS	Degree	2	0
9	Male	37	White	Unemployed	15	20	32	26	71	SSRI (three), SNRI	CS, CBT	Masters	6	0
10	Female	36	Black	Unemployed	8	21	47	28	75	SSRI (two), NSSRI	CS	Left uni	18	3
11	Female	64	White	Employed	15	18	24	16	72	SSRI (four), SNRI (two), NDRI, MAOI, Na + channel blocker, SARI, DRI	CBT	PhD	1	3
12	Male	45	White	Employed	8	21	35	17	68	SSRI, TCA	CBT	Uni	0	0
13	Male	27	White	Employed	7	18	29	26	55	SSRI, TCA, SARI, NDRI	CBT	Masters	8	0
14	Male	49	White	Unemployed	30	23	36	29	70	SSRI (four), SNRI, TCA, NDRI	JA, GT	Degree	0	1
15	Male	56	Black	Unemployed	30	25	44	36	66	SSRI, SARI	CBT	Degree	0	0
16	Male	42	White	Unemployed	22	17	45	29	69	SSRI (three), SARI (two), TCA	None	Degree	0	0
17	Male	31	Asian	Unemployed	6	19	44	20	66	SSRI, SNRI	None	Left school	0	1
18	Male	58	White	Part retired	10	16	28	28	61	SSRI (two), SARI	JA	Degree	0	0
19	Male	62	White	Retired	15	17	42	24	74	SSRI (two), TCA, pregabalin	JA	Masters	15	0
20	Male	44	White	Unemployed	20	14	27	28	68	SSRI (three), SARI, SNRI, Na + channel blocker, TCA, MAOI	CBT, MBT	Degree	20	0
Group	6 females	44.1 (11)	15 White	11 Unemployed	17.7 (8.5)	19 (2.7)	35 (7.4)	23.9 (5.4)	68.5 (6.0)	4.6 (2.6)	17 psychotherapy	18 higher ed	3.7 (6.5)	0.7 (1.1)

Eligible patients attended a pretreatment MRI scan and psychological preparation visit, followed by two dosing sessions, separated by 1 week. In the first session, patients received 10 mg psilocybin and in the second, 25 mg. Patients were seen the following day for debriefing and a post-treatment MRI scan, and for one final time 1 week after the 25-mg session. Subsequent follow-up measures were collected remotely. Patients emailed their completed questionnaires to the study team. Six-month follow-up interviews were carried out by RW with all 20 patients and the relevant qualitative data are reported elsewhere (Watts et al. 2017).

### Reporting Side effects

Side effects were documented based on patient reports in response to the question: “Have you experienced any side effects in relation to the treatment?” This was asked at all post-treatment visits and any spontaneously reported or observed side effects were also documented.

### Psychological support

Psychological support comprised of three components: (1) preparation (P), (2) acute and peri-acute support (S) and (3) integration (I). (1) Preparation (P) involves getting to know the patient and his/her background, building a relationship of trust and providing some information on what can be expected from psilocybin and how best to navigate its effects. (2) Support (S) involves being physically and emotionally present for the patient before, during and after the acute drug session. It may incorporate empathetic listening and reassurance, for example. (3) Integration (I) involves non-judgmental listening to the patient’s testimony after his/her experience and may occasionally feature some interpretation regarding the content of the experience and its potential meaning, as well as advice regarding maintaining and cultivating positive changes in outlook and lifestyle. We assign the acronym PSI to these core components of psychological support.

### 11-Dimension altered states of consciousness (11D-ASC) questionnaire

This is a 94-item questionnaire, of which 44 items are scored. The 44 items are factorised according to a previous validation paper (Studerus et al. 2010). Each item is scored as in a visual analogue scale with the upper anchor reading “much more than usual” and the bottom one reading “no more than usual”. Patients performed the 11D-ASC at the end of each dosing day when the subjective effects of psilocybin had subsided to a negligible level; however, ratings were done with reference to the period when effects were most intense. *t* tests with Bonferroni correction (revised *α* = 0.05/11 = 0.0045) contrasted scores for the 10- and 25-mg dose sessions.

### Data analysis

Two-tailed paired *t* tests were performed for all pre- vs post-treatment QIDS-S16 contrasts, with Bonferroni corrected *α* of 0.05/6 = 0.0083 for the six post-treatment time intervals. 95% confidence intervals (CI) are provided. Effect sizes were calculated using Cohen’s *d* values for dependent data. We chose not to correct for additional clinical measures beyond correcting for QIDS-SR16 changes at multiple time points. This decision was made so as to avoid introducing type 2 errors through overly conservative correction and because of the high covariance between clinical measures (see “Results” section). For transparency, we provide all relevant *p* values and effect sizes.

## Results

### Patients

One hundred and twenty people expressed an interest in the study. Seventy-four were considered appropriate for a telephone screen, from which 29 were invited for a screening visit. Twenty were ultimately recruited for the trial and 19 completed all measures. Data on 12 of the 20 have been previously reported (Carhart-Harris et al. 2016) and these 12 are included in the present analysis. Patients’ demographic and clinical characteristics are shown in Table 1. Eighteen of the 20 patients met the criteria for severe or very severe depression at baseline (QIDS-SR16 score of ≥ 16); the remaining two meeting the criteria for “moderate” depression (QIDS-SR16 score ≥ 11, < 16). The median number of (lifetime) failed previous medications was 4, the mean was 4.6 ± 2.6 and the maximum was 11. The mean duration of illness of the sample was 17.7 ± 8.4 years (range = 7–30 years), as assessed by the question: “For how long has your current depression lasted?” Note that none of the demographic variables were predictive of treatment response, including past use of psilocybin.

**Fig. 1:**
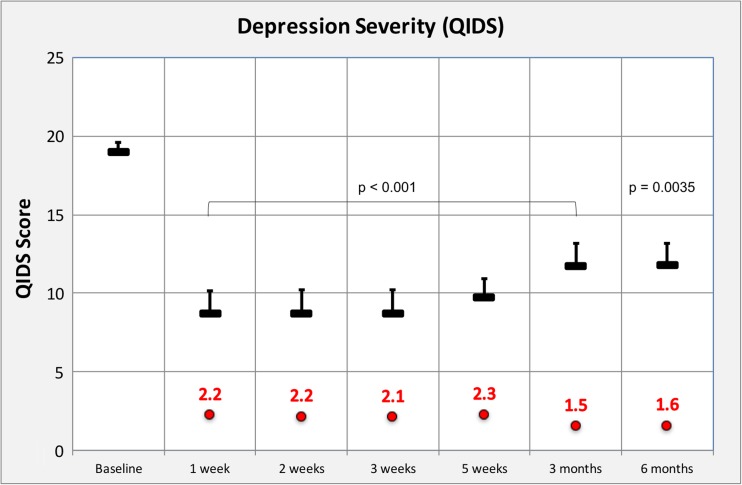
Depression severity vs time: depression severity determined by the primary outcome measure, self-rated QIDS-SR16. Mean values were calculated for the 19 completers. Data are shown for the QIDS scores of 16–20 considered to reflect severe depression. All post-treatment assessments were obtained after the high-dose session, i.e. 1-week post-treatment refers to 1 week after the 25-mg psilocybin dose. Mean values are represented by the black horizontal bars with positive standard errors also included. Cohen’s *d* values vs baseline are shown in red, all contrasts vs baseline yielded *p* values of < 0.001 with the exception of the 6 month contrast which was *p* = 0.0035. Patient 17’s data is not included in the chart due to absent data points at 1 week to 4 months; however, his baseline and 6-month data is included in the text contained in “Results” section and retrospective ratings for 1 and 3 weeks post-treatment were also obtained and are reported in the text only

Data were analysed for the 19 who completed all assessment time points. Relative to baseline, QIDS-SR16 scores were significantly reduced at all six post-treatment time points (*p* < 0.001), with the maximum effect size at 5 weeks (− 9.2, 95% CI = − 11.8 to − 6.6, *t* = − 7.2, *p* < 0.001, Cohen’s *d* = 2.3) (see Fig. 1). Of the 19 patients who completed all assessments, all showed some reduction in depression severity at 1 week and these were sustained in the majority for 3–5 weeks. Changes in HAM-D ratings from baseline to 1-week post-treatment showed a reasonable correspondence with changes in QIDS-SR16 data across the same period (*r* = 0.61, *p* < 0.001) and the relationship between the QIDS-SR16 and BDI at 1 week was very strong (*r* = 0.81, *p* < 0.001).

BDI scores were significantly reduced at 1 week (mean reduction = − 22.7, 95% CI = − 17.6 to − 27.8, *p* < 0.001), 3 months (mean reduction = − 15.3, 95% CI = − 8.7 to − 21.9, *p* < 0.001) and 6 months post-treatment (mean reduction = − 14.9, 95% CI = − 8.7 to − 21.1, *p* < 0.001); STAI-T anxiety scores were significantly reduced at 1 week (mean reduction = − 23.8, 95% CI = − 16.5 to − 31.1, *p* < 0.001), 3 months (mean reduction = − 12.2, 95% CI = − 6.1 to − 18.3, *p* < 0.001) and 6 months post-treatment (mean reduction = − 14.8, 95% CI = − 8.1 to − 21.6, *p* < 0.001); SHAPS anhedonia scores were significantly reduced at 1 week (mean reduction = − 4.6, 95% CI = − 2.6 to − 6.6, *p* < 0.001) and 3 months post-treatment (mean reduction = − 3.3, 95% CI = − 1.1 to − 5.5, *p* = 0.005); HAM-D scores were significantly reduced at 1 week post-treatment (mean reduction = − 14.8, 95% CI = − 11 to − 18.6, *p* < 0.001); and GAF scores were significantly increased 1 week post-treatment (mean increase = + 25.3, 95% CI = 17.1 to 33.5, *p* < 0.001)—see Table 2.

**Table 2:** Individual patient clinical ratings: clinical outcomes at various time points. The clinician administered ratings were completed at baseline and 1 week post-dosing only

	BDI				STAI				SHAPS			HAM-D		GAF	
	Baseline	1 week	3 months	6 months	Baseline	1 week	3 months	6 months	Baseline	1 week	3 months	Baseline	1 week	Baseline	1 week
Mean (SD)	34.5 (7.3)	11.8 (11.1)	19.2 (13.9)	19.5 (13.9)	68.6 (6.1)	44.8 (15.7)	56.5 (13.3)	53.8 (13.3)	6.6 (4.1)	1.9 (2.7)	3.3 (4.2)	24.1 (5.4)	9.3 (7.6)	48.9 (10.3)	74.2 (16.05)
Difference vs baseline (SD)		− 22.7 (10.6)	− 15.3 (13.7)	− 14.9 (12.0)		− 23.8 (15.2)	− 12.2 (12.7)	− 14.8 (14)		− 4.6 (4.1)	− 3.3 (4.6)		− 14.8 (7.8)		+ 25.3 (17.1)
Cohen’s *d*		2.5	1.4	1.4		2.2	1.2	1.5		1.3	0.8		2.3		1.9
*p* value		*p* < 0.001	*p* < 0.001	*p* < 0.001		*p* < 0.001	*p* < 0.001	*p* < 0.001		*p* < 0.001	*p* = 0.005		*p* < 0.001		*p* < 0.001

Treatment was generally well tolerated and there were no serious adverse events. One patient became uncommunicative during the peak of his 25-mg psilocybin experience but this normalised after the acute drug effects had abated. Follow-up discussions revealed that his experience had been “blissful” and beneficial but also overwhelming (see supplementary file). Regretfully, this patient chose not to complete further follow-up measures, with the exception of the QIDS-SR16 and BDI scores at 6 months post-treatment. Follow-up scores were 25 (QIDS) and 40 (BDI) at 6 months. See Watts et al. (2017) for more details about individual cases.

A brief note: this experience, combined with evidence supporting the importance of patient-therapist rapport in the psychedelic treatment model (e.g. Carhart-Harris et al., in review), has motivated us to revise the exclusion criteria for future psilocybin trials, i.e. with “psychiatric condition judged to be incompatible with establishment of rapport with therapy team and/or safe exposure to psilocybin, e.g. suspected borderline personality disorder” added as a criterion for exclusion.

Consistent with our earlier report on the initial 12 patients from this trial (Carhart-Harris et al. 2016), transient anxiety lasting for minutes (*n* = 15) and headaches lasting no more than 1–2 days (*n* = 8) were the most common side effects. Five reported transient nausea but there were no cases of vomiting. Three reported transient paranoia within the duration of the acute drug experience but this was short-lived in every case. As with all our previous work with this compound, there were no reported cases of so-called flashbacks or persisting perceptual changes.

Fourteen patients reported visions of an autobiographical nature. In most cases, such visions were regarded as insightful and informative. One patient reported a vision of his father attempting to physically harm him when he was child, something he claimed not to have been previously conscious of. This patient subsequently felt confused about the authenticity of this putative memory and this was associated with a transient worsening of symptoms (see weeks 2 and 3 in fig. S1). Appealing to clinical equipoise, the study team felt it best practice not to make a judgement on the veridicality of this alleged memory but open and compassionate listening was maintained and the patient subsequently improved.

Suicidality scores on the QIDS-SR16 were significantly reduced 1 and 2 weeks post-treatment (mean reductions at week 1 = − 0.9, 95% CI = − 0.4 to − 1.4, *p* < 0.002; mean reduction at week 2 = − 0.85, 95% CI = − 0.4 to − 1.3, *p* = 0.004), with trend decreases at 3 (mean reduction = − 0.8, 95% CI = − 0.25 to − 1.3, *p* = 0.01) and 5 weeks (mean reduction = − 0.7, 95% CI = − 0.22 to − 1.2, *p* = 0.01). Scores on the suicide item of the HAM-D were significantly decreased 1-week post-treatment (mean reduction = − 0.95, 95% CI = − 0.58 to − 1.3, *p* < 0.001), with 16 of 19 patients scoring 0 at this time point and none showing an increase from baseline nor scoring the maximum on this measure. Scores on the genital/sexual dysfunction item of the HAM-D were also significantly reduced 1-week post-treatment (mean reduction = − 0.58, 95% CI = − 0.18 to − 0.98, *p* = 0.002) and no one scored the maximum nor showed an increase in sexual dysfunction from baseline.

The complete 11D-ASC scores can be found in the supplementary file. After Bonferroni correction (0.05/11 = 0.004), values for *experience of unity* (mean difference = 0.26, 95% CI = 0.12 to 0.41, *p* = 0.001), *spiritual experience* (mean difference = 0.28, 95% CI = 0.11 to 0.41, *p* < 0.001), *blissful state* (mean difference = 0.3, 95% CI = 0.16 to 0.44, *p* < 0.001), *insightfulness* (mean difference = 0.26, 95% CI = 0.11 to 0.41, *p* < 0.001) and *complex imagery* (mean difference = 0.18, 95% CI = 0.08 to 0.28, *p* < 0.001) were found to be significantly higher after 25 mg psilocybin than the 10-mg dose.

**Fig. 2:**
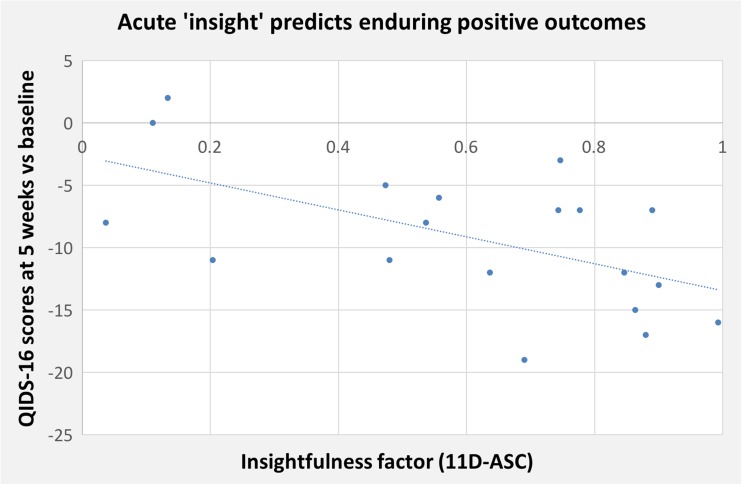
Acute ‘insight’ measured by the ‘insightfulness’ factor of the 11D-ASC rated in the evening after the 25-mg psilocybin experience correlated significantly with reductions in depressive symptoms 5 weeks later (*r* = − 0.57, *p* = 0.01, two-tailed)

Previous work has indicated a strong relationship between the following 11D-ASC factors: experience of unity, spiritual experience and blissful state (Studerus et al. 2010); and a multiple correlation analysis confirmed their inter-relatedness here (*r* > 0.92 for all permutations). We therefore decided to treat them as one factor (assigned the acronym ‘USB’), taking mean values for each patient. Testing the hypothesis that this *USB* factor and *insight* would predict better clinical outcomes, we found significant relationships between mean scores of USB and insight (Fig. 2) during the 25-mg psilocybin experience and changes in QIDS-SR16 scores at 5 weeks (*r* = − 0.49, *p* = 0.03 and *r* = − 0.57, *p* = 0.01, respectively).

After the 6-month endpoint, information was collected on other treatments received by the patients (Watts et al. 2017). With the exception of patient 2 (who remained on venlafaxine throughout the trial and also received CBT shortly afterwards), no patients received additional treatments within 5 weeks of the 25-mg psilocybin dose. Six began new courses of antidepressant medication after the 3-month time point. Five received psychotherapy (CBT, psychodynamic, counselling and group therapy × 2) shortly before or after the 3-month period and five sought and successfully obtained psilocybin (without sanction from the study team) between 3 and 6 months. Removing the five that obtained psilocybin from the 3- and 6-month analyses did not substantially alter the main results: at 3 months, the effect size increased to 1.6 and the *p* value remained < 0.001; and at 6 months, the effect size increased to 1.7 and the *p* value became 0.018.

Assessing relapse at 6 months in responders (at 5 weeks) revealed only three of nine cases—with the remaining six maintaining response—even when using conservative criteria for relapse of QIDS score of 6+ or above at 6 months. These data tentatively imply that psilocybin may protect against relapse to an equivalent extent to daily use of an established antidepressant—as seen in discontinuation trials where responders either continue on medication (33% relapse) or transfer to placebo (46% relapse) for 6 months (Gueorguieva et al. 2017). Two major caveats here, however, are that one cannot reliably extrapolate from a sample of nine, and whereas patients in our trial received no interventions from us beyond the integration work done 1 week after their 25-mg psilocybin session, patients in clinical trials typically ingest a potentially active antidepressant daily for 6 months.

## Discussion

This paper presents updated and extended data from an open-label clinical trial assessing psilocybin with psychological support for treatment-resistant depression. Findings corroborate our (Carhart-Harris et al. 2016) and others’ previous results (Griffiths et al. 2016; Ross et al. 2016; Grob et al. 2011) supporting the safety and efficacy of psilocybin for depressive and anxiety symptoms. A fast and sustained response exceeding what might be expected from a placebo response was observed in many of the patients (see Carhart-Harris and Nutt (2016) for a relevant discussion). Notably, *all* 19 completers showed some reductions in the QIDS-SR16 scores at 1-week post-treatment and (nominally) maximal effects were seen at 5 weeks. Other interventions, not formally part of the present trial, confounded outcomes at 3 and 6 months, although safety was maintained and a sizeable proportion of the sample continued to demonstrate benefit (see Watts et al. (2017) for more details). Conclusions on efficacy are limited by the absence of a control condition in this trial, however.

Recent studies (Griffiths et al. 2016; Ross et al. 2016; Carhart-Harris et al. 2016), including the present one, help demonstrate the feasibility of treating patients with major depressive disorder with psilocybin plus psychological support. Two recent double-blind randomised control trials (RCTs) of psilocybin for depression and anxiety symptoms in a combined sample of 80 patients with life-threatening cancer found consistent safety and efficacy outcomes with those reported here (Griffiths et al. 2016; Ross et al. 2016). Only a subset of patients recruited into these studies met the criteria for major depressive disorder however, and symptoms were not of the same severity as those seen here (i.e. mean baseline BDI scores were 18.1 and 16 in the Griffiths et al. and Ross et al. studies, respectively, whereas they were 35 in the present study). A comprehensive RCT designed to properly assess psilocybin’s efficacy for major depressive disorder, with some form of placebo control, is therefore warranted (Carhart-Harris and Goodwin 2017).

Regarding mechanisms, we recently proposed a model by which psychedelic-induced 5-HT2AR signalling rapidly induces an acute state of plasticity in which an enriched context (Carhart-Harris et al., in review) may lead to cognitive biases being revised (Carhart-Harris and Nutt 2017; Carhart-Harris and Goodwin 2017)—see also Branchi (2011). The above-reported correlation between acute ‘insightfulness’ and enduring reductions in depressive symptoms may be viewed as broadly supportive of this model. Moreover, recently published fMRI data collected as part of the present trial may help to develop and refine this model (Roseman et al., in review; Carhart-Harris et al., in review).

Future research should endeavour to better characterise, control and measure the various psychological components contained within the current psychedelic treatment model. There is an assumption that individuals under the influence of a psychedelic are especially sensitive to the *context* in which the experience occurs, both in terms of (1) prior expectations and other relevant state and trait factors and (2) environmental factors, e.g. the quality of the relationships with persons attending to them before, during and after the experience and patients’ relationship to the music listened to during the sessions (Kaelen et al. 2015)—and this matter has recently been discussed in length (Carhart-Harris et al., in review). In order to properly assess the relative contribution of these variables and their assumed interactions with psilocybin, it will be necessary to properly control and measure them, and this has presently not yet been done to a satisfactory level (see Carhart-Harris et al. (in review) for suggestions on how this might be done).

Relatedly, psychotherapeutic models used to support and mediate the psilocybin experience need to be better defined, tested and potentially manualised. Basic principles for safe therapeutic work with psychedelics can be found in guidelines (Johnson et al. 2008) and books (Richards 2015) but more systematic verification, refinement and (eventual) manualisation of treatment approaches are needed for subsequent roll-out (Carhart-Harris and Goodwin 2017). Moreover, cost-effectiveness will become increasingly salient as the development of psilocybin as a treatment model progresses. The major qualifier here is that experiments intended to evaluate the contribution of psychological variables to the psychedelic experience need to be conceived and conducted with an appreciation of the special vulnerability of individuals under the influence of psychedelics (again, see Carhart-Harris et al. (in review). Thus, certain standards of care, including a certain level of psychological support, may be non-negotiable if safety is to be maintained.

An obvious limitation of the present study is its open-label design and absence of a control condition. The initial plan was to conduct a placebo-controlled RCT but regulatory and drug procurement challenges meant that available resources could only support a smaller trial. The present results may be viewed as a successful demonstration of proof-of-principle, however, supporting the view that psilocybin can be given safely, even in severe cases of depression, with the caveat that appropriate control of context (e.g. the provision of psychological support and a comfortable environment) is essential for positive outcomes (Carhart-Harris et al., in review). Impressions of efficacy gleaned from the present study’s findings may be cautiously described as ‘promising’—and if supported by larger and better controlled trials, psilocybin’s low toxicity, favourable side effect profile and putative rapid and enduring antidepressant action could render it at least competitive with currently available treatments for major depression, whose therapeutic actions may be either delayed, e.g. in the cases of SSRIs and psychotherapy, or short-lived, e.g. in the case of ketamine. Comparative efficacy trials may therefore be an interesting next step. Such designs may also have merit in terms of addressing the challenge of maintaining the study blind in trials with psychedelics (Carhart-Harris and Goodwin 2017).

Another limitation of the present trial is that the final eight patients were all male. This is regretful as it limits extrapolation to the general population, where rates of treatment-resistant depression may be marginally higher in women than in men (Kubitz et al. 2013). Greater effort will be made in future trials to recruit more representative samples of the target population. Another limitation deserving of mention is the issue of assessing duration of current depressive episode. Patients gave estimates based on the question “For how long has your current depression lasted?” but some chose to estimate based on the duration of their chronic illness, believing they had not experienced a discernable remission for years–decades, even during periods when their symptoms were relatively less severe.

In summary, we have presented updated and extended data from a feasibility trial assessing psilocybin with psychological support for treatment-resistant depression. With the caveat that this was an open-label trial with no control condition, safety and efficacy outcomes continue to support the case for further research (Carhart-Harris and Goodwin 2017). Identifying key psychological and pharmacological variables comprising the treatment model, and testing their assumed interactions, is one of a number of important next steps (Carhart-Harris et al., in review).

## Electronic supplementary material

ESM 1 (DOCX 6136 kb)
