# Supplementary material for: Psilocybin with psychological support for treatment-resistant depression: six-month follow-up
Source: Psychopharmacology (Berl). 2017 Nov 8;235(2):399–408. doi: 10.1007/s00213-017-4771-x (PMC5813086; doi:10.1007/s00213-017-4771-x)
Supplement: Supplementary file 1 — (DOCX 6136 kb) [file 213_2017_4771_MOESM1_ESM.docx]

**Supplementary material**

**11D-ASC scores in full:**

11D-ASC scores for 10mg psilocybin: *experience of unity* (0.34 ± 0.31), *spiritual experience* (0.33 ± 0.26), *blissful state* (0.33 ± 0.29), *insightfulness* (0.36 ± 0.3), *disembodiment* (0.44 ± 0.3), *impaired cognition* (0.25 ± 0.22), *anxiety* (0.24 ± 0.28), *complex* *imagery* (0.47 ± 0.34), *elementary* *imagery* (0.49 ± 0.42), *audio*/*visual* *synesthesia* (0.52 ± 0.38), *meaning* (0.3 ± 0.26).

For the 25mg experience, scores were as follows: *experience* *of* *unity* (0.59 ± 0.36), *spiritual* *experience* (0.62 ± 0.36), *blissful* *state* (0.64 ± 0.32), *insightfulness* (0.6 ± 0.3), *disembodiment* (0.61 ± 0.31), *impaired* *cognition* (0.33 ± 0.26), *anxiety* (0.35 ± 0.31), *complex* *imagery* (0.66 ± 0.31), *elementary* *imagery* (0.62 ± 0.35), *audio*/*visual* *synesthesia* (0.65 ± 0.39), *meaning* (0.47 ± 0.3).

**Individual scores on the QIDS-SR16**


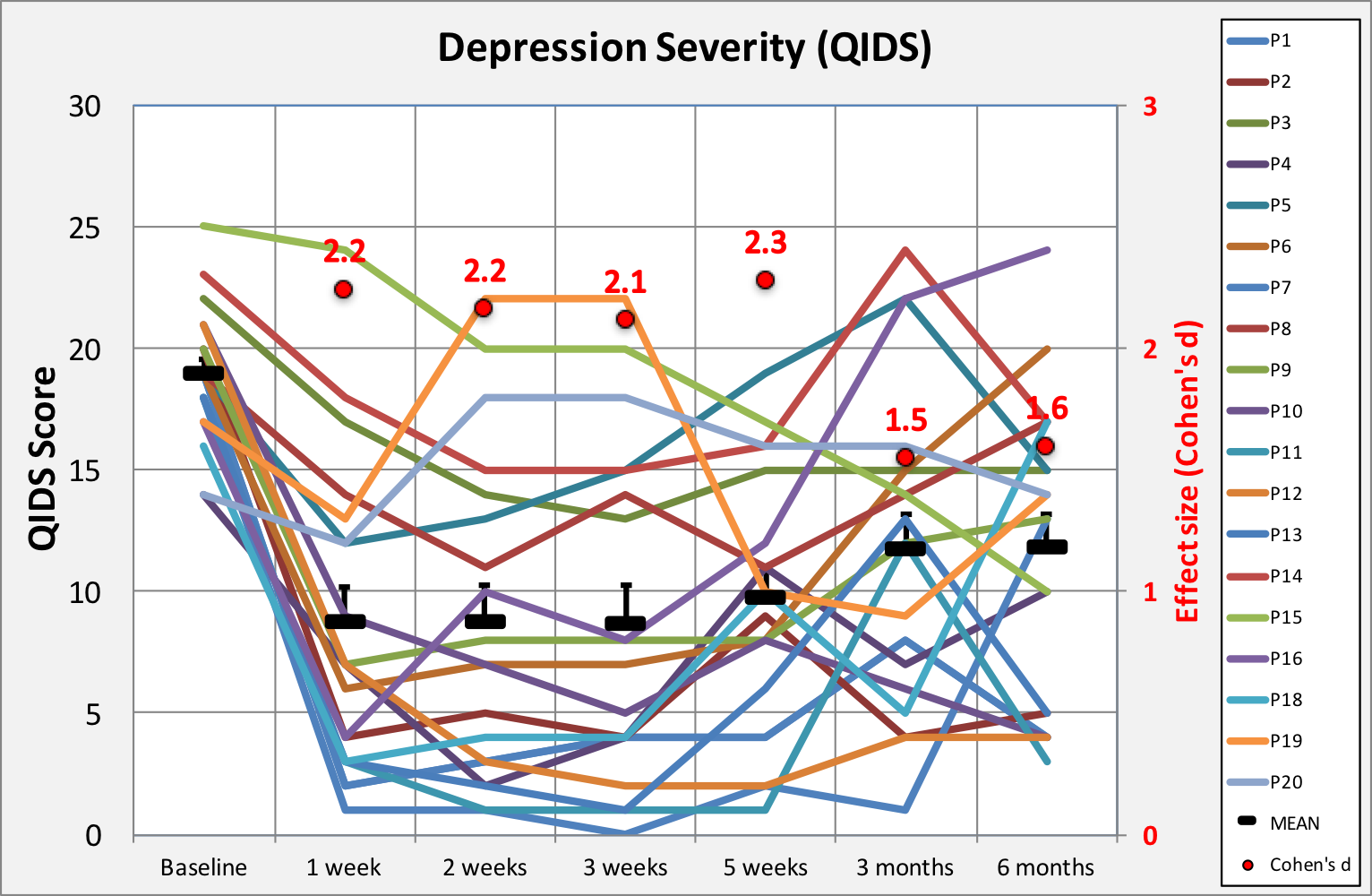


**Figure S1. Depression severity vs time:** Depression severity determined by the primary outcome measure: self-rated QIDS-SR16. Mean values were calculated for the 19 completers. Data are shown for the QIDS scores of 16-20 are considered to reflect severe depression, 11-15 moderate, 6-10 mild and 5 and below = no depression. All post-treatment assessments were obtained after the high-dose session, i.e. 1-week post-treatment refers to 1 week after the 25mg psilocybin dose. Mean values are represented by the black horizontal bars with positive standard errors also included. Cohen’s d values vs baseline are shown in red, all contrasts versus baseline yielded p values of < 001 with the exception of the 6 month contrast which was p = 0.0035. Patient 17’s data is not included in the chart due to absent data-points at 1-week to 4-months; however, his baseline and 6-month data is included in the text contained in the results section and retrospective ratings for 1 and 3 weeks post-treatment were also obtained and are reported in the text only.

**Patient who chose not to complete follow-up clinical measures: transcript from 6-month interview performed by RW (see Watts et al. 2017) for more information:**

**About the second dose:**

Patient: “At some point in the second dosing I must have thought of something which triggered me having a kind of, I guess I’d describe it as sexual bliss, something, prolonged orgasmic, climactic and also eternal. It didn’t have a gender, it had a humanistic connection, I was connected to other human beings. I’m normally heterosexual; but this feeling was connected to both male human beings and female human beings and it wasn’t connected to physical attributes it was more connected to, I guess you could describe it as their souls. And um the sexual feeling also didn’t have a feeling connected to my body, sexual organs, it was something above that, like a bliss something beyond what you feel, everyone feels that alongside orgasm, part is connected to sexual organs, and another subtle part alongside that is a glowing blissful feeling that is beyond the physical And it was just that feeling on its own, but really intensely, it was connected to male and female souls, all ages, not their physical attributes, it included even old people, it wasn’t connected to a sexual act or thought of a sexual act, it was different souls, and connecting to these souls resulted in this blissful feeling, it got more intense, really overwhelming, the glow grew until I couldn’t sense the specific things about humans, there was no space for thought I was just that sexual feeling and nothing else, so I didn’t have consciousness, I was not human, I had become bliss.”

**Interviewer: “How long did [the benefits] last for?”**

Patient: “A few months ago I still got the feeing that I’m a lot better and it was definitely the trial, but there are loads of things I haven’t finished, things I’ve been failing at, my memory, I’m so frustrated so its difficult to connect to the feeling of freedom I had just a couple of months ago: it’s blurred out now.”

**Interviewer: “How would you describe that feeling of relief?”**

“I don’t know its just… it’s a feeling that I’m not depressed and I don’t have this depression and collective problems, I don’t feel them - I feel free of them, and then I think ‘hang on have I ever been depressed?’ and then I think maybe this trial helped me feel that way.”

**Interviewer: “If you were to compare it to other drugs?”**

Patient: “It ranks very highly; I would be keen on taking it again.”

**Interviewer: “In an ideal world, when would you have wanted another dose?”**

“I wish I had it back when I first started having serious mental health problems back in 2001, it would have prevented all this damage, I think using psilocybin in the beginning would have prevented all that damage.”

**Reference**

Watts RD, C; Krzanowski, J; Nutt, D, J; Carhart-Harris, R, L (2017) Patients' accounts of increased 'connection' and 'acceptance' after psilocybin for treatment-resistant depression. *Journal of Humanistic Psychology.*
